# Silk micrococoons for protein stabilisation and molecular encapsulation

**DOI:** 10.1038/ncomms15902

**Published:** 2017-07-19

**Authors:** Ulyana Shimanovich, Francesco S. Ruggeri, Erwin De Genst, Jozef Adamcik, Teresa P. Barros, David Porter, Thomas Müller, Raffaele Mezzenga, Christopher M. Dobson, Fritz Vollrath, Chris Holland, Tuomas P. J. Knowles

**Affiliations:** 1 Department of Chemistry, University of Cambridge, Lensfield Road, Cambridge CB2 1EW, UK; 2 Department of Materials and Interfaces, Weizmann Institute of Science, Rehovot 76100, Israel; 3 Department of Health Science and Technology, ETH Zurich 8092, Switzerland; 4 Department of Zoology, University of Oxford, South Parks Road, Oxford OX1 3PS, UK; 5 Department of Materials Science and Engineering, University of Sheffield, Sheffield S1 3JD, UK; 6 J.J. Thomson Avenue, Cavendish Laboratory, University of Cambridge, Cambridge CB3 0HE, UK

## Abstract

Naturally spun silks generate fibres with unique properties, including strength, elasticity and biocompatibility. Here we describe a microfluidics-based strategy to spin liquid native silk, obtained directly from the silk gland of *Bombyx mori* silkworms, into micron-scale capsules with controllable geometry and variable levels of intermolecular β*-*sheet content in their protein shells. We demonstrate that such micrococoons can store internally the otherwise highly unstable liquid native silk for several months and without apparent effect on its functionality. We further demonstrate that these native silk micrococoons enable the effective encapsulation, storage and release of other aggregation-prone proteins, such as functional antibodies. These results show that native silk micrococoons are capable of preserving the full activity of sensitive cargo proteins that can aggregate and lose function under conditions of bulk storage, and thus represent an attractive class of materials for the storage and release of active biomolecules.

## Abstract (web summary)

Silk fibres currently used in biotechnology are chemically reconstituted silk fibroins (RSF), which are more stable than native silk fibroin (NSF) but possess different biophysical properties. Here, the authors use microfluidic droplets to encapsulate and store NSF, preserving their native structure.

The control of protein denaturation and aggregation is the cornerstone of successful silk production^1^. In silk glands of arthropods, this control is achieved through a tightly regulated phase transition of the component proteins from a soluble, largely disordered random coil structure into a solid fibrous form consisting of ordered intermolecular hydrogen bonded β*-*sheet rich aggregates^2^. Native silk produced by the *Bombyx mori* silkworm consists mainly of two proteins, native silk fibroin (NSF) and sericin, with NSF providing the structural core of the silk fibers and sericin providing a coating layer^1^. A detailed understanding of the phase transition process that results in fiber formation is not only of great academic interest, but also provides key information for any successful attempt to mimic the exceptional material properties observed in natural silk^1^,^3^,^4^,^5^,^6^,^7^. Many components of fibrillar silk can be chemically re-solubilized; in particular reconstituted silk fibroin (RSF)^5^,^6^, obtained by dissolving spun cocoons, has been used in a wide range of applications.^5^,^6^,^8^,^9^,^10^,^11^,^12^,^13^,^14^,^15^ The widespread use of reconstituted silk feedstocks is largely enabled by the relative ease in which this material can be prepared and stored^16^. NSF feedstocks can be obtained directly from the gland of the silkworm where it is stored in a spatially distinct location from sericin^3^. However, such feedstocks are renowned for their extreme sensitivity to shear and high propensity to aggregate once isolated^17^, in marked contrast to RSF, which is significantly more stable in solution^1^,^17^,^18^,^19^. Indeed, the inherent difficulties in handling NSF feedstocks significantly limit the potential of this material for use in biotechnological applications.

To address the fundamental challenges in processing NSF, we have explored a microfluidic platform that enables the investigation of the artificial spinning of native silk as well as providing routes towards its long-term storage in an active state ready for a range of possible uses. We report the generation of a wide range of micron-scale shapes, herein referred to as micrococoons or micron-scale capsules^20^,^21^, and demonstrate that morphological diversity can be generated through fine tuning of the shear conditions, and through variation in the flow rates used, and by modulating the surface tension and viscosity of the feedstock. Importantly for potential applications, we find that micrococoons exhibit a distinct core-shell structure with an internal environment apparently ideal for the storage of sensitive and aggregation-prone materials.

## Results

### Micron scale silk capsule synthesis

**Figure 1:**
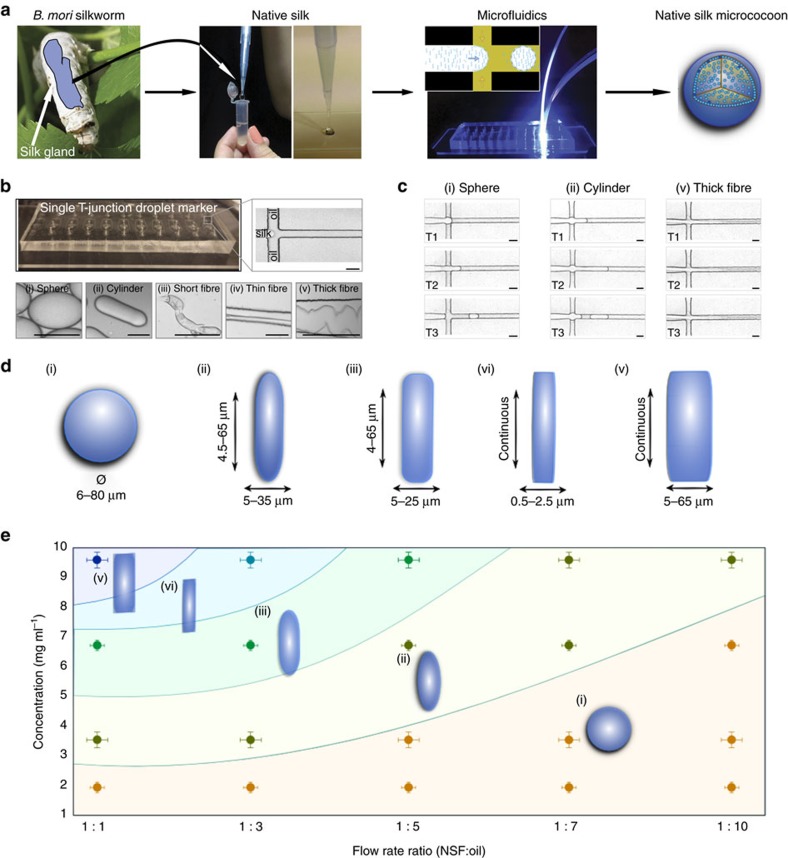
Micrococoon synthesis. (**a**) Schematic representation of the microfluidic processing of NSF into micrococoons. (**b**) Optical microscopy images of the NSF micrococoons formed at a single T-junction in the microfluidic device. Micrographs of a variety of NSF micrococoon shapes are shown in the lower panels: (i) sphere, (ii) cylinder, (iii) short fibre, (iv) thin fibre, (v) thick fibre. Scale bar, 20 μm. (**c**) Micrographs of NSF micrococoon formation acquired at three different time points T1, T2 and T3: (i) sphere; T1=0 ms, T2=10 ms, T3=13 ms, (ii) cylinder; T1=0 ms, T2=13 ms and T3=34 ms and (v) thick fibre; T1=0 ms, T2=59 ms, T3=68 ms. Scale bar, 20 μm. (**d**) S schematic representations showing the characteristic dimensions of the NSF micrococoons generated in this study. (**e**) Different micrococoon shapes generated as a function of the protein concentration and of the ratio of the flow rates of the aqueous to oil phases.

We have applied a microfluidic strategy to control the level of shear applied to an NSF solution^22^,^23^ to induce the transition of NSF from its initial, native state, into highly aggregated β*-*sheet-rich silk microstructures formed as monodispersed microemulsions^24^ (Fig. 1a). NSF micrococoons were synthesized at a T-junction in a microfluidic device (Fig. 1b,c) by co-flowing an immiscible fluorinated oil phase from both sides of a stream of NSF in the central microchannel (see Methods). The instability of the aqueous stream towards breaking up into droplets leads to the generation of a highly monodisperse silk-in-oil emulsion at the T-junction. Moreover, the shear experienced during the co-flow leads to the formation of gelled material as the NSF converts into its aggregated state at the aqueous/oil interface where the shear is greatest (see Methods and Supplementary Fig. 1a,b). By controlling the viscosity of the NSF feedstock and the shear rate (Fig. 1e and Supplementary Fig. 1a) we were able to form a variety of different micron-scale morphologies, including spherical and cylindrical structures as well as continuous fibres (Fig. 1d,e). We also discovered that the range of morphologies could be further increased by introducing a second T-junction in series with the first one on the same microfluidic chip. In this manner, the NSF micrococoons produced at the first T-junction could be encapsulated by a second layer of NSF to form double shell structures leading to more intricate forms of structure (Supplementary Fig. 1c,d).

The final shape of the micron-scale NSF capsules was found to be determined by a combination of the concentration and viscosity of the NSF feedstock and the relative flow rates of the aqueous and the oil phases (Fig. 1e). The viscosity of NSF solutions increased with increasing protein concentration^14^, and elongated micrococoons were observed to be the dominant structure formed at concentrations above 5 mg ml^−1^. By contrast, when the NSF concentration was lowered to below 2 mg ml^−1^, spherical microdroplets were the major species formed. These observations suggest a dominant effect on the morphology of surface tension at low concentrations and viscosities, favouring a minimal interface area and hence a spherical shape, and of shear forces at high concentrations and viscosities (Supplementary Fig. 1a,b), leading to structures with high aspect-ratios such as cylinders and fibres.

**Figure 2:**
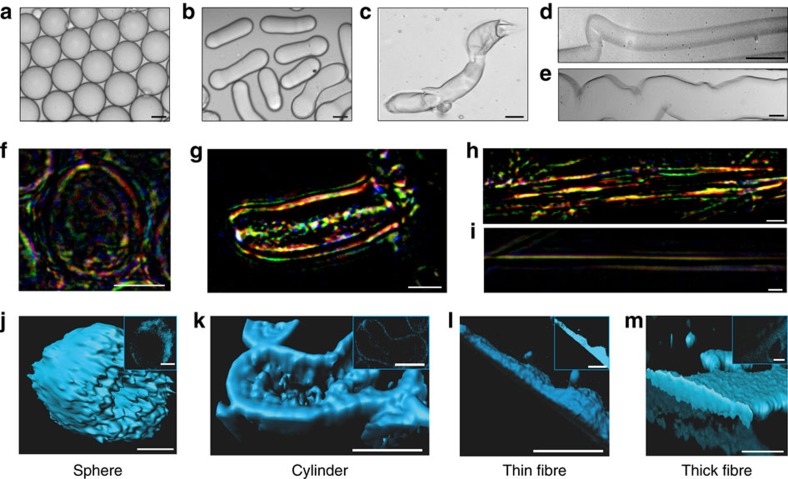
Micrococoon morphology. Bright field microscopy images of NSF micrococoons: (**a**) spheres, (**b**) cylinders, (**c**) short fibres, (**d**) thin fibres and (**e**) thick fibres. Scale bar, 5 μm. Images of micrococoon structures placed between crossed polarizers: (**f**) sphere, (**g**) cylinder, (**h**) thick fibre, (**i**) thin fibre. Scale bar, 10 μm. 3D reconstructions of confocal images are shown for: (**j**) a sphere, (**k**) a cylinder, (**l**) a thin fibre, (**m**) a thick fibre. The z-stack central cut images are shown in the inserts. Scale bar, 10 μm.

The efficiency of the conversion of NSF into micron-scale capsules (Fig. 2a–e) was studied by using UV spectroscopy and colorimetric methods (see Methods) to establish the concentration of unconverted NSF remaining in solution after isolation of the micrococoons. For all micrococoon shapes, the observed conversion efficiency was very high, reaching values of 87±3%. Moreover, the loss of 13±3% of NSF protein can be attributed simply to the partial disassembly of a small fraction of micron-scale capsules during the washing steps used to transfer the micrococoons from the continuous oil phase, used in the emulsification process, to an aqueous environment (see Methods).

### Micrococoon morphology

The dependence of the surface morphology on the different micrococoon shapes was studied by atomic force (AFM; Supplementary Fig. 2a–e) and confocal microscopy (Fig. 2j–m). The spherical micrococoons exhibited smooth surfaces (Supplementary Fig. 2a), while the elongated structures were observed to contain aligned nano-scale wrinkles (Supplementary Fig. 2b–e). For the cylindrical structures, these surface features were oriented parallel to the long axis of the micrococoons (Supplementary Fig. 2b), while for the fibril-like structures they were oriented perpendicular to the fibre axis, an observation likely to originate from mechanical stresses acting on the gelled outer shell (Supplementary Fig. 2c–e) under the compressive stresses present in the microfluidic channel.

We also monitored the alignment of the silk nanofibrils in the micrococoon shapes by probing their interaction with linearly polarized light. We observed that NSF in solution does not alter the polarization of light, and such samples remained dark when placed between crossed polarizers (Supplementary Fig. 2f). By contrast, fibers spun naturally by silkworms, which consist of aligned fibroin fibrils coated with sericin, exhibit birefrigent behaviour, with the optical axis aligned along the axis of the fibril, as shown in Supplementary Fig. 2g, and consistent with previous literature reports^25^,^26^,^27^. We then proceeded to probe in this manner the microcapsules generated in this work. The birefrigence images shown in Fig. 2f–i reveal a high level of alignment, in particular within the shells of the spherical micrococoons, and along the length of the more elongated capsule structures. The multitude of colours observed in Fig. 2f–h may originate from variations in the thickness of the NSF fibrillar layer, and a non-uniform distribution of soluble and fibrillar NSF in the micrococoons, as well as to a wavelength-dependence of the refractive index^28^, and to a lesser degree the absorption coefficient.

### Structural changes during microcapsule formation

The transformation of NSF from its initial disordered structure to highly ordered β*-*sheet-rich aggregates was also followed by monitoring the changes in fluorescence that accompany this transition^29^. The maximum emission intensity of the intrinsic blue fluorescence signal for the NSF aggregates was in the range of 425 to 450 nm, with a small variation attributed to the biological diversity of the silkworms from which the silk was obtained. This characteristic spectral shift allowed aggregated NSF to be detected and localized spatially through confocal microscopy. The results shown in Fig. 2j–m reveal an accumulation of aggregated NSF on the outside of the micron-scale capsules where the shear forces during formation were largest.

**Figure 3:**
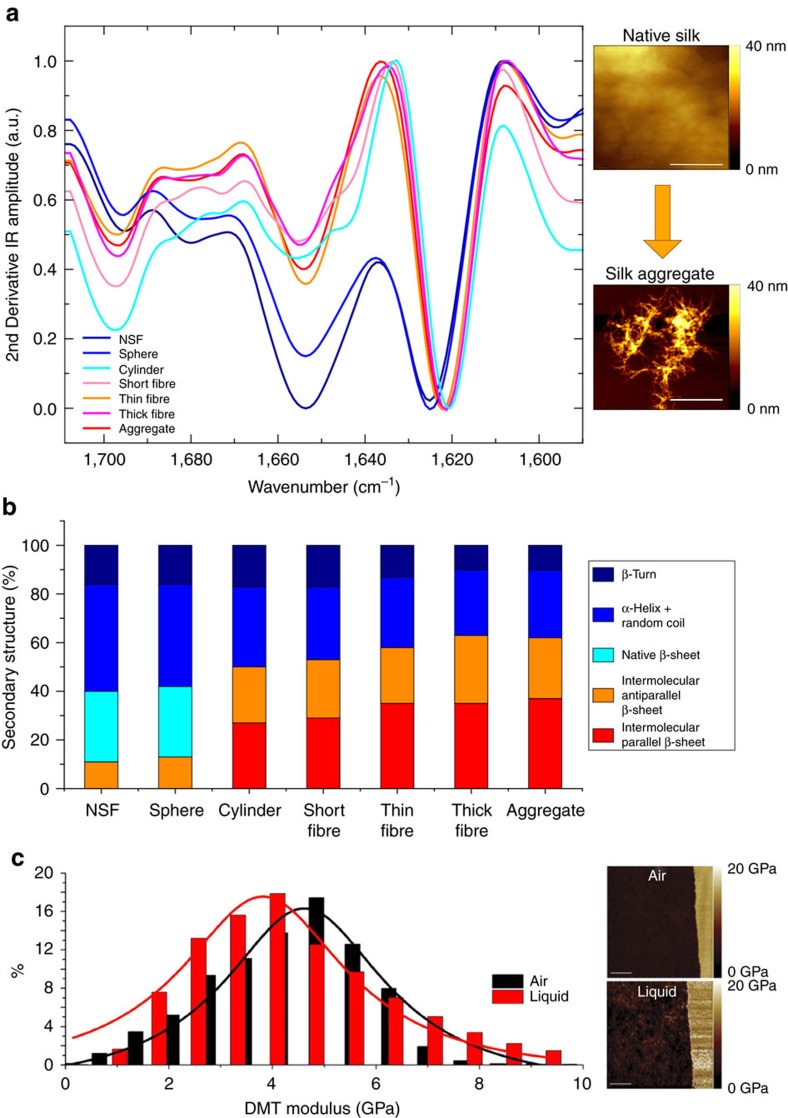
Ultrastructural properties of micrococoon. (**a**) FTIR spectra of spheres, cylinders and of, short, thin and thick fibres. AFM images of native and aggregated NSF are shown at the right hand side of the FTIR spectra with scale bars of 400 nm. (**b**) Chart summarizing the structural changes of NSF upon its conversion into its different forms, calculated from the amide I bands in the FTIR spectra (Supplementary Fig. 3a). (**c**) AFM DMT modulus (Derjaguin-Muller-Toporov model of elastic contact) images in air and in liquid (right) with the corresponding distribution of the DMT modulus (left). Scale bar, 3,000 nm.

The structural changes in NSF during conversion into micrococoon shapes were also examined using Fourier transform infrared (FTIR) spectroscopy. The FTIR spectrum, in particular in the region containing the characteristic protein amide I bands, is highly sensitive to the secondary and tertiary structure content of proteins^30^,^31^,^32^,^33^,^34^,^35^. The results show that the soluble NSF is predominantly disordered (Fig. 3a,b) with minor contributions from α*-*helical, β*-*turn and random coil structures, in agreement with literature reports on this material^7^,^25^,^36^,^37^. In some samples, we observed a small contribution also from inter-molecular β*-*sheet structure; however, we cannot completely exclude the possibility that a low level of inter-molecular β*-*sheet could be formed during the sample preparation for FTIR analysis. The aggregated state by contrast (Supplementary Fig. 3a) is rich in intermolecular β*-*sheet structure with a high degree of order, indicating the formation of a dense network of intermolecular hydrogen bonds. The formation of these intermolecular contacts further differentiates spectroscopically the aggregate state from the native one that is dominated by intramolecular hydrogen bonds within native intramolecular β*-*sheets. This difference is in particular apparent in the shift of the amide band I towards lower wavenumbers, characteristic of the formation of the intermolecular hydrogen bonds.

The differences in the morphologies of the different types of micrococoons were found to correlate with differences in the relative abundances of native intra-molecular versus inter-molecular β*-*sheet secondary structure indicative of the presence of aggregated silk fibrils. In particular, the spherical morphologies were found to have ∼30% of native β*-*sheet structure, a value that is similar to that of soluble NSF, while the filamentous micron-scale capsules have ∼60\% of inter-molecular β*-*sheet structure (Fig. 3b; Supplementary Fig. 3b,c), largely absent from soluble NSF. These results are in good agreement with polarized microscopy observations (Fig. 2f–i) that indicate pronounced formation of fibrils in the elongated forms of the micrococoons as well as with confocal microscopy data (Fig. 2j–m), which show that these structures possess a fully aggregated core. The process of NSF micrococoon formation suggests an explanation for these differences, as the spherical micron-scale capsules are formed under conditions of low shear; the NSF therefore gels only at the surfaces of the droplets, and in the interior of the structures it remains in its soluble native form and is encapsulated by a thin shell of aggregated NSF. With increasing shear rates, the formation of fibrillar structure becomes more extensive, resulting, in the case of the elongated capsules, in the complete conversion of the NSF into its structurally ordered aggregated form.

### Micrococoon elastic properties

We next probed the Young’s modulus of the NSF micron-scale capsules by means of AFM nanoindentation and peak force quantitative nanomechanical mapping (PF-QNM), both in air and in liquid^38^,^39^. Figure 3c and Supplementary Fig. 2h show representative DMT-modulus AFM images with corresponding distributions of the DMT modulus and three-dimensional (3D) topographic AFM images with corresponding height profiles, of a spherical micron-scale capsule in air and in liquid. The average Young’s modulus of the shell was measured to be 4.6 GPa in air and 3.8 GPa in liquid, respectively (Fig. 3c and Supplementary Fig. 2h) values, which are consistent with β*-*sheet rich materials^40^,^41^.

### Protein storage and release

**Figure 4:**
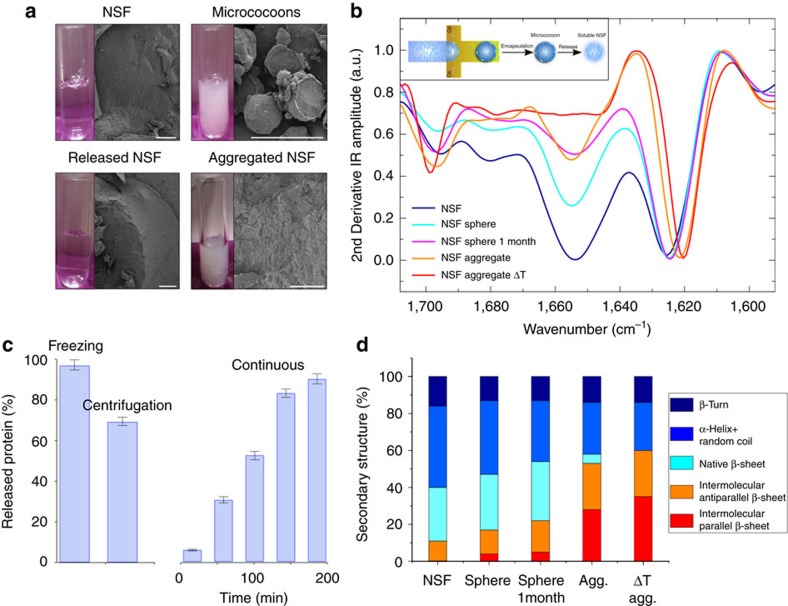
NSF long-term storage and release. (**a**) Cryo-SEM micrographs showing the morphology of (from left to right) soluble NSF, NSF micrococoons, released and aggregated NSF. Scale bars, 100 μm; The corresponding images of NSF solutions are shown as inserts (left); (**b**) FTIR spectra of the aggregation and release of NSF by snap-freezing in liquid nitrogen. Insert: schematic representation of the formation of spherical NSF micrococoons and NSF release from micrococoons; (**c**) histogram showing the efficiency of NSF release by snap-freezing, gentle centrifugation and continuous washing of the NSF micrococoons. The indicated error bars are the s.d. of the average of three different repeats, each one measure 12 times. (**d**) Histogram of NSF secondary structure elements calculated from the amide I band in the FTIR spectra (**b**) after release from the microncapsules.

We have shown how microfluidic processing allows NSF to be transformed into micrococoons with a degree of aggregation that can be tuned to give rise either to shells containing soluble NSF or fully aggregated micrococoons. We next explored the potential of the long-term encapsulation within the formed structures of aggregation-prone NSF for storage and subsequent controllable release. We synthesized a macroscopic volume of spherical NSF micrococoons, each of which consisted of a thin shell with a thickness of 1–2 μm, as shown in Fig. 4a,b, with the rest of the volume of the micrococoon consisting of soluble NSF accumulated within the shell. Under bulk conditions, the soluble form of NSF is stable for only a few hours at room temperature, but within the micron-scale capsules, is was found to retain its native properties for at least one month (Fig. 4b,d). Furthermore, even after storage for this length of time, the majority of the liquid NSF could be released from the micrococoons by rupturing the outer shells by means of low temperatures or increased hydrostatic pressure generated by centrifugation, Fig. 4b–d. We monitored this release process by measuring the concentration of soluble protein by UV absorption; the results revealed that 98% of the NSF could be recovered from the micrococoons by snap freezing in liquid nitrogen (Fig. 4c). Release by ultracentrifugation allowed ∼80% of the protein to be recovered, with ∼20% being lost through aggregation during the release process as a consequence of the mechanical shear encountered during the centrifugation step. The soluble NSF stored for 1 month was found by FTIR (Fig. 4b,d) to be extremely similar in structure to that observed prior to storage. Indeed, the high level of protection offered by the encapsulation of NSF within our micrococoons was shown by the observation that the NSF released from such structures could in turn be reconverted into micrococoon shells and stored again, a process that was successfully repeated at least five times with the same starting material.

To probe further the stabilizing effect confered on soluble NSF within the micrococoons, we monitored the structural changes of the silk protein solution in bulk and when stored in the gelled micrococoons as a function of environmental parameters such as mechanical shear, temperature and ionic strength. We first exposed NSF samples in both bulk and encapsulated forms to mechanical stress by continuous shaking, and followed the conformational changes by FTIR spectroscopy (Supplementary Fig. 4). The results show almost complete transformation of the NSF into β*-*sheet-rich aggregates within only 20 s (Supplementary Fig. 4a,b) under bulk conditions, while almost no changes were observed in the secondary structure of the NSF encapsulated inside micrococoons (Supplementary Fig. 4c,d) after 1 h of continuous shaking. We next exposed both systems to elevated temperatures and high ionic strengths. The results are shown in Supplementary Fig. 5a–d, and demonstrate that the soluble NSF stored in bulk converted into its fibrous form within a few seconds, as shown in Supplementary Fig. 6a–d, while NSF inside the micrococoons remained unaffected. Taken together, these data demonstrate that the silk micrococoons protect the encapsulated NSF from a range of factors promoting aggregation.

**Figure 5:**
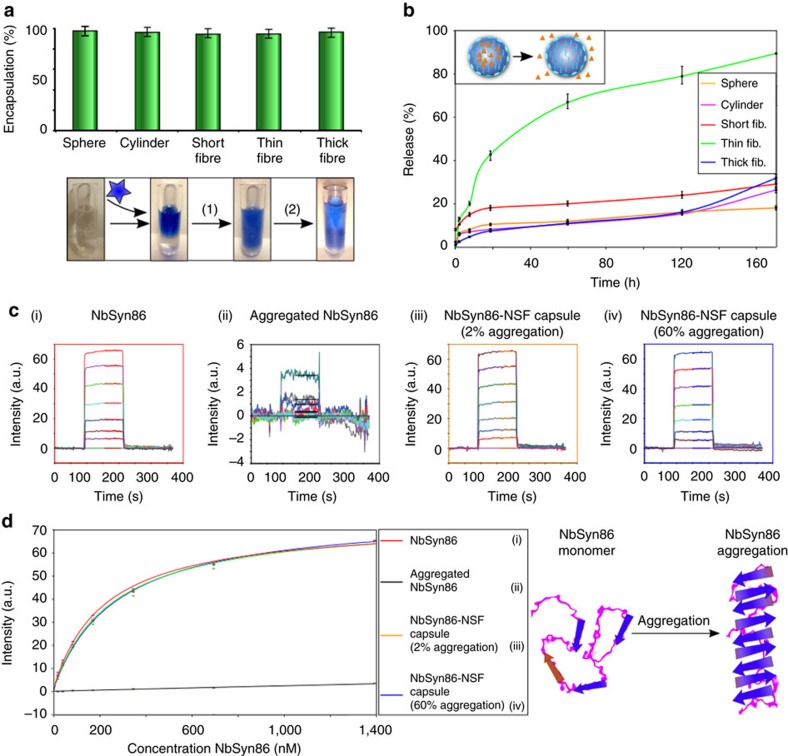
Antibodies stabilization by encapsulation. (**a**) Encapsulation efficiency studies for the C4scFv single chain Fv domain in spheres, cylinders, short, thin and thick fibres. The indicated error bars are the s.d. of the average of three different repeats, each one measure 10 times. Insert: schematic representation of the encapsulation and release of antibiody domains by NSF micrococoons: step (1) encapsulation, step (2) release. (**b**) Release kinetics for C4scFv from different NSF micrococoon shapes. The indicated error bars are the s.d. of the average of three different repeats, each one measure 10 times. (**c**) Biacore sensorgrams of the binding of NbSyn86 to immobilized α-synuclein: (i) a control sample of monomeric NbSyn86, (ii) NbSyn86 after encapsulation and release treatment in the absence of NSF, (iii) NbSyn86 released from gelled NSF micrococoons (which contain ca. 2% of aggregated NSF), (iv) NbSyn86 released from gelled micrococoons which contain ca. 60% of aggregated NSF. (**d**) Graph of the equilibrium binding values for the different released NbSyn86 samples (as shown in **c**) versus the initial (pre-encapsulation) concentration of NbSyn86. The data were fitted to a 1:1 bimolecular binding model to estimate the affinity constant of NbSyn86 for α-synuclein, and were also used to estimate the loss of activity between samples. The indicated error bars are the s.d. of the average of three different repeats, each one measure five times.

The ability of NSF micrococoons to provide long-term storage for NSF itself suggested their potential use for the stabilization of other aggregation sensitive protein species. Antibodies provide an important example of proteins that often possess a high propensity to aggregate, a factor that can limit significantly their efficacy and shelf-life. We examined the encapsulation, stabilization and release of several active antibody species, including a single-chain Fv-binding domain specific for the protein huntingtin, C4scFv (ref. 42), and two single chain Fv domains specific for α*-*synuclein, NbSyn86 and NbSyn87 (ref. 43). First, we tested the encapsulation and release efficiencies by using C4scFv antibodies labelled with AlexaFluor647 (See Methods). We achieved very high loading efficiencies (>95%, Fig. 5a) as well as efficient and rapid release kinetics (Fig. 5b), without any loss of binding activity or solubility (Supplementary Fig. 9). Next, we probed the effect of the NSF micrococoons on the stability of one of the domains, NbSyn86, which had previously been shown to have relatively low thermal stability in bulk solution^44^ (Fig. 5c, ii) and a high propensity to self-aggregate resulting in a significant reduction of its binding activity. The results, shown in Fig. 5 and Supplementary Fig. 9, demonstrate that the binding activity of C4scFv, NbSyn86 and NbSyn87 before and after encapsulation and release was identical. Moreover, the micrococoons demonstrated their ability to enhance markedly the stability of the aggregation-prone single chain Fv domain (See Fig. 5c, iii–iv); in bulk solution, NbSyn86 rapidly aggregated when heated to 65 °C, while identical heat treatment of the domain encapsulated in silk micrococoons led to no measurable loss of activity (Fig. 5c,d). These results suggest a route towards the development of effective stabilization systems for storage of highly sensitive functional macromolecular species.

In addition, we investigated the potential of the micrococoons to serve as transporters of small molecules (Supplementary Fig. 7a), a possibility that is particularly interesting for drug delivery in view of the biocompatibility of silk (Supplementary Fig. 8) in biomedical applications^7^,^45^,^46^. We found that the NSF micrococoons have an outstanding ability to encapsulate, store and release in a controlled manner small molecules, including glucose and the antibiotic tetracycline, under physiological conditions, as we show in the Supporting Information (Supplementary Fig. 7). Moreover, the micrococoons were found to be non-toxic to human cells (Supplementary Fig. 8) and, therefore, are likely to have the potential as novel safe delivery vehicles for external as well as for internal use.

## Discussion

In conclusion, the complex rheology of NSF and its tendency to aggregate presents a significant challenge for the storage and processing of this potentially highly functional material. Our results, however, show that multiphase flow of NSF in microfluidic systems can be used to overcome these limitations through the flexible processing of NSF into a wide range of micron-scale capsules. We have found that such NSF micrococoons have the ability to provide a practical solution for the long-term storage and control of NSF feedstocks, with an increase in stable storage time of several orders of magnitude relative to conventional storage under bulk conditions. In addition, we have demonstrated that NSF micrococoons can be used to encapsulate other sensitive molecules, such as functional antibodies, in a way that offers significant protection against their aggregation and loss of function^12^. Protein molecules, including antibodies, are increasingly used in therapeutic applications, but often posses a high tendency to undergo unwanted aggregation processes and lose function. The ability of silk micrococoons to control and curtail this behaviour should, therefore be of considerable significance for the long-term storage of proteins in functional states.

## Methods

### Micrococoon preparation

The following materials were used for preparation of NSF micrococoons: freshly extracted NSF from the *B. mori* silkworm gland^3^, fluorinert FC-70 (Sigma-Aldrich, UK) and N,N bis (*n*-propyl)polyethylene oxide-bis(2-trifluoromethyl polyperfluoroethylene oxide) amide surfactant^47^.

### Droplet microfluidics

The single and double T-junction droplet makers were fabricated from PDMS (polydimethylsiloxane, ca. 50,000∼Mw, Sylgard 184, Dow Corning, USA) as chips by using standard soft lithography methods^48^, ^49^, ^50^, ^51^. The synthesis of the NSF micrococoons was performed on a specially designed microfluidic system with 20 μm diameter channels. 1 ml of aqueous NSF at pH7 and 1 ml of fluorinert oil containing 2% w/v of N,N′bis(n-propyl)polyethylene oxide-bis(2-trifluoromethyl polyperfluoroethylene oxide) amide surfactant, were mixed at the T-junctions of microfluidic channels by using flow control through syringe pumps. The initial concentration of NSF varied from 1 to 10 mg ml^−1^. The multi-shell micron-scale capsules were formed using a double T-junction device in which the NSF and oil solutions were mixed at the first T-junction to form their initial shape and then passed through the second T-junction with NSF dope as a continuous phase. The capsules were then washed with doubly distilled water (DDW) at pH7 to remove the surfactant and any unreacted protein.

### Confocal and light microscopy

Samples were deposited as aqueous dispersions, without further purification, onto a glass slide. The NSF micrococoons were analysed by confocal microscopy (Laser Scan Confocal, Zeiss Microscope 5,100), using a laser 405 nm at 25 mW for violet excitation. Because of the intrinsic native fluorescent signal emitted from the aggregated NSF protein, the NSF micrococoons were analysed by confocal microscopy without labelling; the emission maxima, in the blue region of the fluorescence spectrum, originated from the aggregated component of the gelled NSF micrococoons, while for the single shell structures the aggregated NSF content was detected at the interface of each micron-scale capsule. The double shell structures exhibited blue emission from the internal as well as the external shells of the NSF micrococoon shapes. 3D images were reconstructed using the ‘Imaris’ image analysis program (on average 412 *z*-stack slices per each protein shell).

### Measurements of loading capacity and release kinetics

To calculate the efficiency of the conversion of the NSF into micrococoons, the concentration of unreacted NSF was measured (after washing) by UV absorption by using a NanoDrop 2,000 UV spectrophotometer (Thermo Scientific, UK) and using a bicinchoninic acid (BCA) protein detection kit (ThermoFisherScientific), following absorption at 562 nm; in no case did the difference between the two approaches exceed 3%. In addition, the loading efficiency and release profiles of the C4scFv (ref. 52) antibody domain from NSF micrococoons were probed using an AlexaFluor647 labelled domain^43^. The loaded micrococoons were washed with PBS at intervals of time from 10 min to 30 days, and the solutes after each washing were analysed by UV and fluorescence spectroscopy.

### Data availability

The data that support the findings of this study are available from the corresponding author upon reasonable request.

## Additional information

**How to cite this article:** Shimanovich, U. *et al*. Silk micrococoons for protein stabilisation and molecular encapsulation. *Nat. Commun.* **8,** 15902 doi: 10.1038/ncomms15902 (2017).

**Publisher’s note**: Springer Nature remains neutral with regard to jurisdictional claims in published maps and institutional affiliations.

## Supplementary Material

Supplementary Information
