# Supplementary material for: Silk micrococoons for protein stabilisation and molecular encapsulation
Source: Nat Commun. 2017 Jul 19;8:15902. doi: 10.1038/ncomms15902 (PMC5524934; doi:10.1038/ncomms15902)
Supplement: Supplementary Information [file ncomms15902-s1.pdf]

SI GUIDE

Type of file: pdf
Size of file: 2,468 KB
Title of file for HTML: Supplementary Information
Description: Supplementary Figures, Supplementary Notes and Supplementary Methods.

Supplementary Information

Supplementary Note

NSF micrococoons were synthesised using a microfluidic droplet maker device by forming micron sized droplets at a T-junction of aqueous and oil phases. The creation of the five basic shapes (sphere, cylinder, short, thin and thick fibres) was achieved by passing NSF dopes through a continuous oil phase, whereby the viscosity of the NSF solution determines the final shape of the resulted NSF droplets. The variety of the NSF shapes was expanded (doubled) by introducing a second T-junction into the microfluidic droplet maker. The conversion of the NSF protein into β–sheet aggregates was followed by changes in its fluorescent properties.

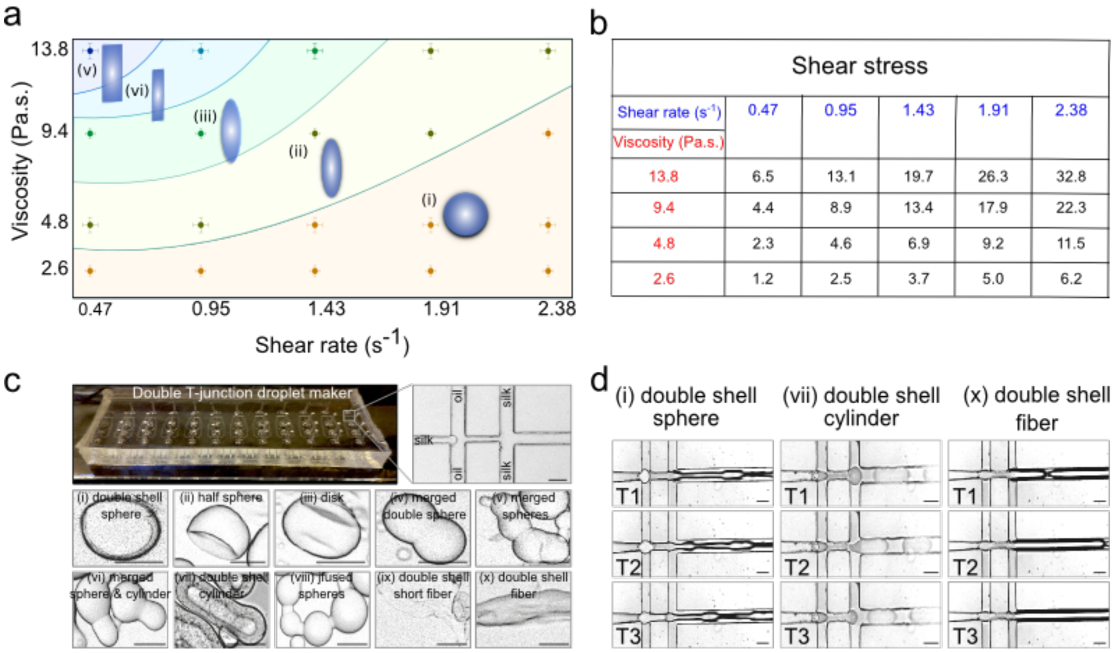

Shear stress					
Shear rate (s^{-1}) / Viscosity (Pa.s.)	0.47	0.95	1.43	1.91	2.38
13.8	6.5	13.1	19.7	26.3	32.8
9.4	4.4	8.9	13.4	17.9	22.3
4.8	2.3	4.6	6.9	9.2	11.5
2.6	1.2	2.5	3.7	5.0	6.2

Supplementary Figure 1. (a) Schematic representation of NSF droplet shape generation as a function of aqueous protein solution viscosity and shear rates calculated from[14]. (b) Calculations of shear stress as a function of NSF viscosity and shear rates based upon data from[14]. (c) Light microscopy images of the NSF micrococoon structures formed in a double T-junction device. The Upper left image shows a photograph of the double T-junction device with a bright field micrograph of the T-junction (right). The following light microscopy images of NSF micrococoon structures are shown: (i) double shell sphere, (ii) half sphere, (iii) disk, (iv) merged double spheres, (v) merged multiple spheres, (vi) merged sphere and cylinder, (vii) double shell cylinder, (viii) fused multiple spheres, (ix) double shell short fibre, (x) double shell thick fibre. Scale Bars are 20 µm. (d) Microscopy images of NSF micrococoon formation acquired at three different time points T1, T2 and T3: (i) double shell sphere; T1 = 0~ms, T2 = 3 ms, T3 = 6 ms, (vii) double shell cylinder; T1 = 0 ms, T2 = 12 ms, T3 = 17 ms and (x) double shell thick fibre; T1 = 0 ms, T2 = 15 ms, T3 = 19 ms. Scale Bars are 20 µm.

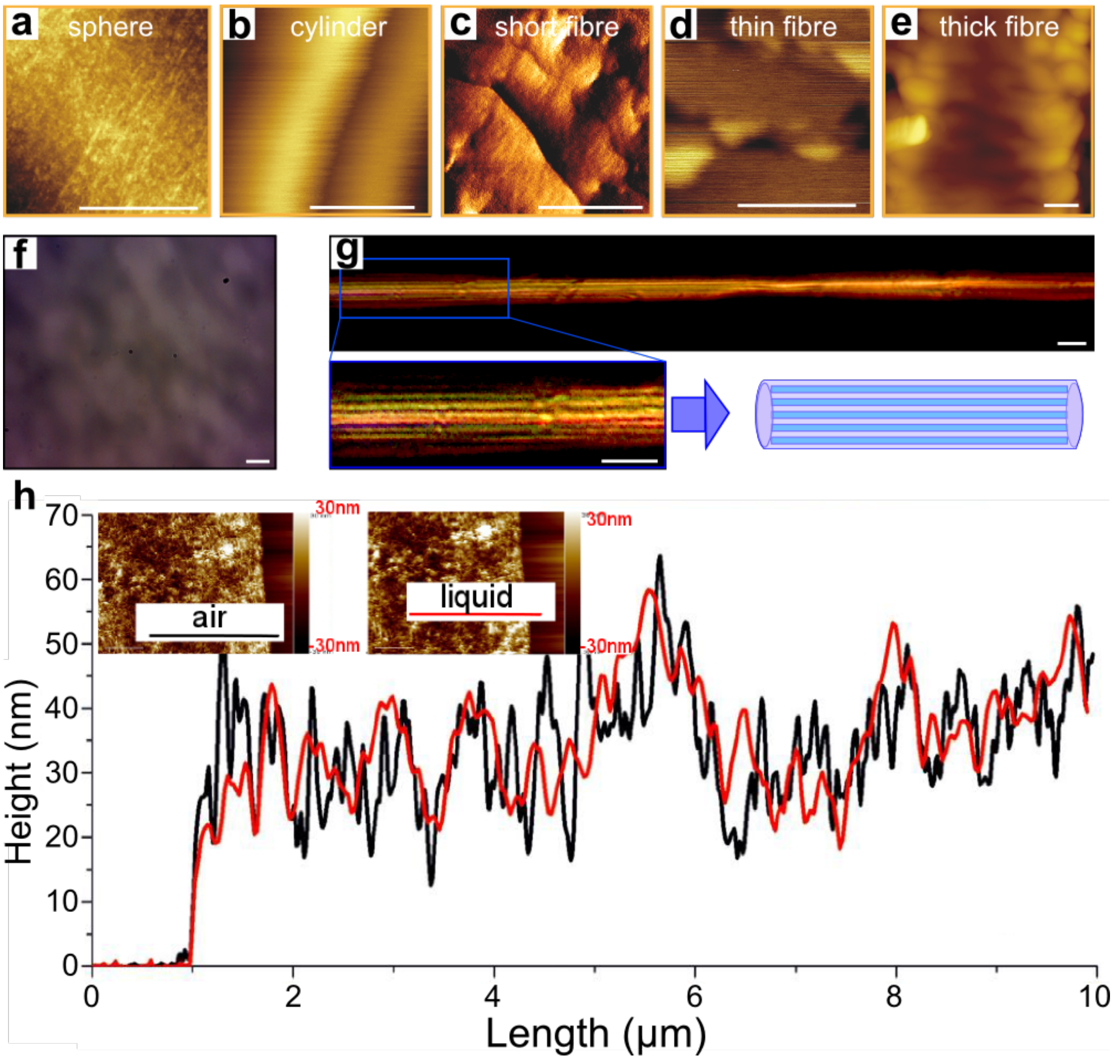

Supplementary Figure 2. Atomic force microscopy images of NSF micrococoon surfaces in the case of: (a) a spheres, (b) a cylinders, (c) a short fibres, (d) a thin fibres and (e) a thick fibres. The scale bars are 300 nm. Images of soluble NSF (f) and NSF fiber spun by the silkworm (g) placed between crossed polarizers and a λ/4 wave plate. (g) (right bottom panel) schematic figure showing the nanofibrils orientation inside the NSF fiber. Scale Bars are 10 µm. (h) AFM nanoindentation studies of spherical micrococoons in air and in liquid. AFM 3D topography images in air and in liquid with corresponding height profiles are shown as an insert. The scale bars are 3000 nm.

a

Band position (cm^{-1})	Assignment
1610-1625	Intermolecular ß-sheet
1620-1635	Native ß-sheet
1635-1648	Random Coil
1650-1660	α-helix
1665-1685	ß-turn
1690-1705	Antiparallel amyloid ß-sheet

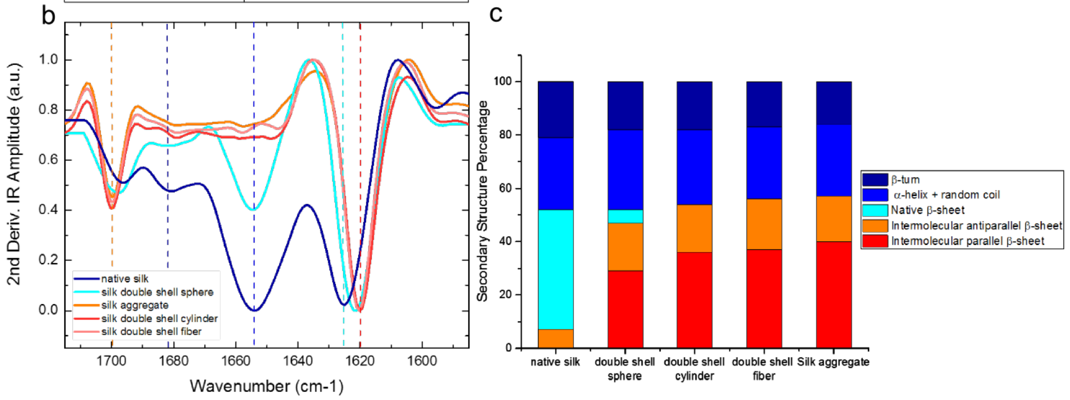

Supplementary Figure 3. (a) Table summarising FTIR band assignment. (b) FTIR spectra of NSF solution, double shell NSF sphere, double shell cylinder and double shell thin fibre shapes. (c) Chart summarising structural changes of NSF protein upon its conversion into the variety of shapes. The calculations were made based on the difference in the amide I peak intensities of FTIR spectra (a)

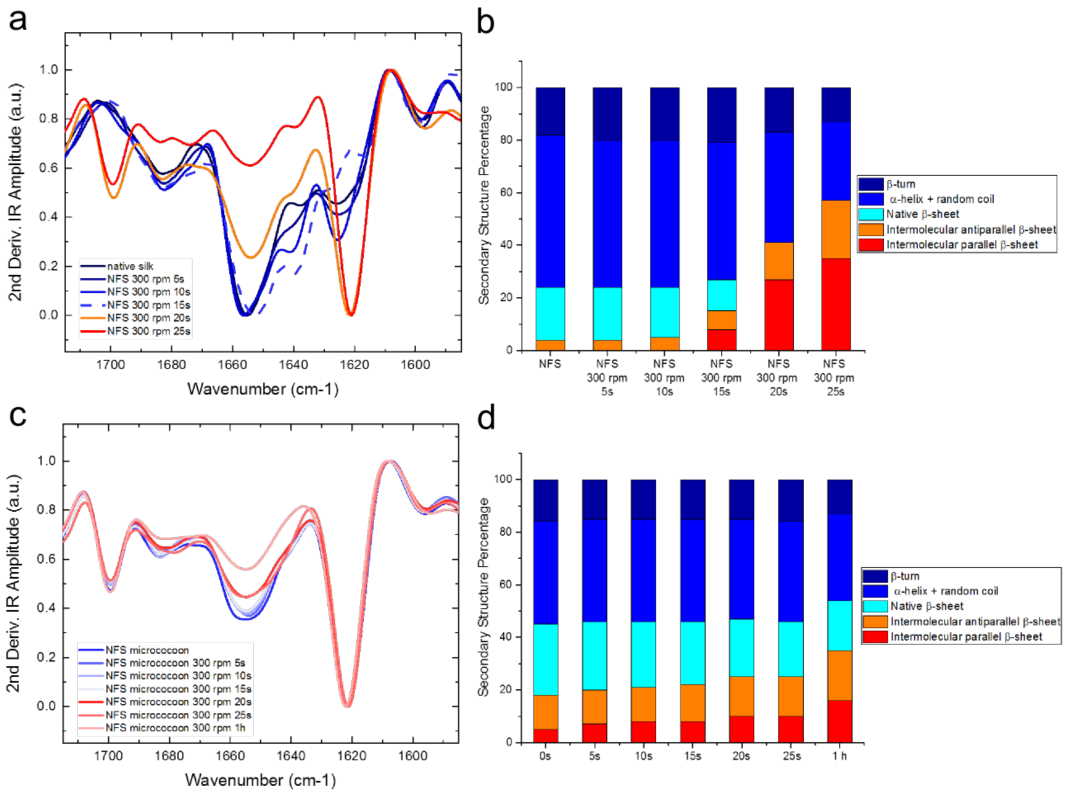

Supplementary Figure 4. (a) FTIR spectra indicating structural changes in NSF exposed to shaking; (300 rpm) for 0 s, 5 s, 10 s, 15 s, 20 s, and 25 s. (b) Chart summarising calculations based on the changes in amide I FTIR spectra (a) of the NSF fraction (in %) that retained its native confirmation after shaking. (c) FTIR spectra indicating structural changes in

encapsulated NSF exposed to shaking; (300 rpm) for 0 s, 5 s, 10 s, 15 s, 20 s, 25 s, 1 h and 12 h. (d) structural changes in encapsulated NSF exposed to shaking.

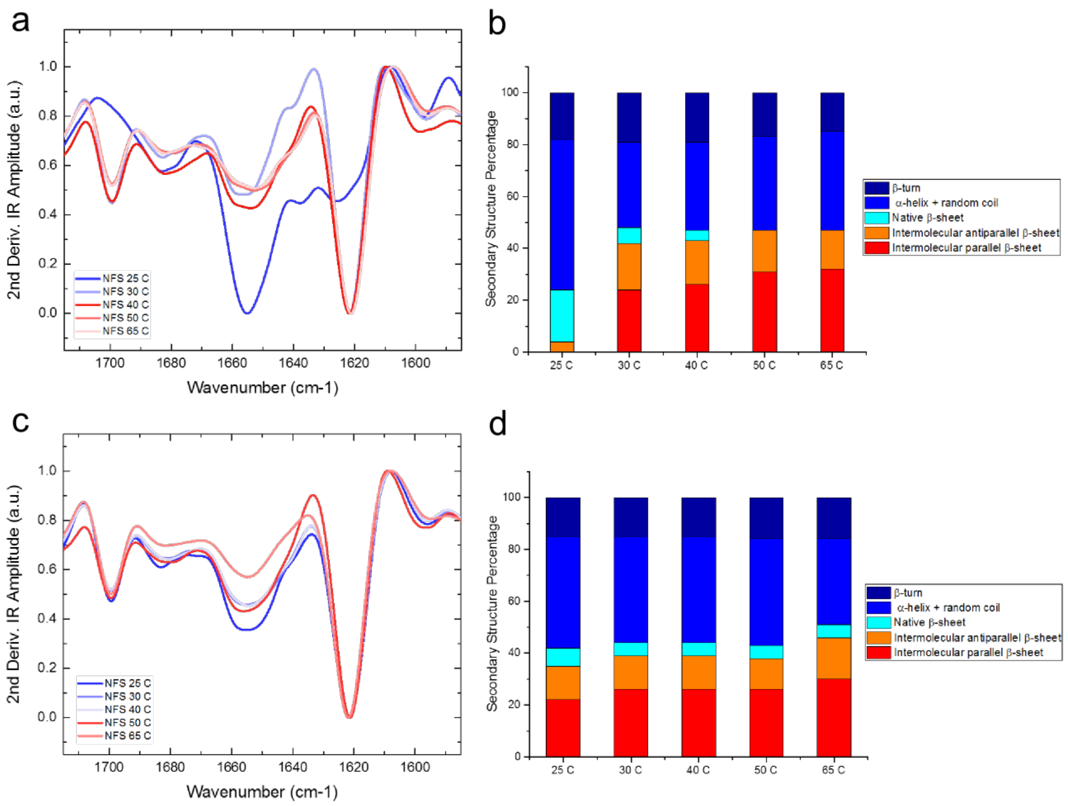

Supplementary Figure 5. (a) FTIR spectra indicating structural changes in NSF exposed to elevated temperatures; 25°C, 30°C, 40°C, 50°C and 65°C. (b) Chart summarising calculations of the NSF fraction (in \%) that retained its native confirmation under elevated temperatures. (c) FTIR spectra indicating structural changes in encapsulated NSF exposed to elevated temperatures; 25°C, 30°C, 40°C, 50°C, 65°C and 65°C for 1h. (d) Structural changes in encapsulated NSF exposed to elevated temperatures. The calculations were made based on the difference in the amide I peak intensities in FTIR spectra from Supplementary Figure 5a and 5c.

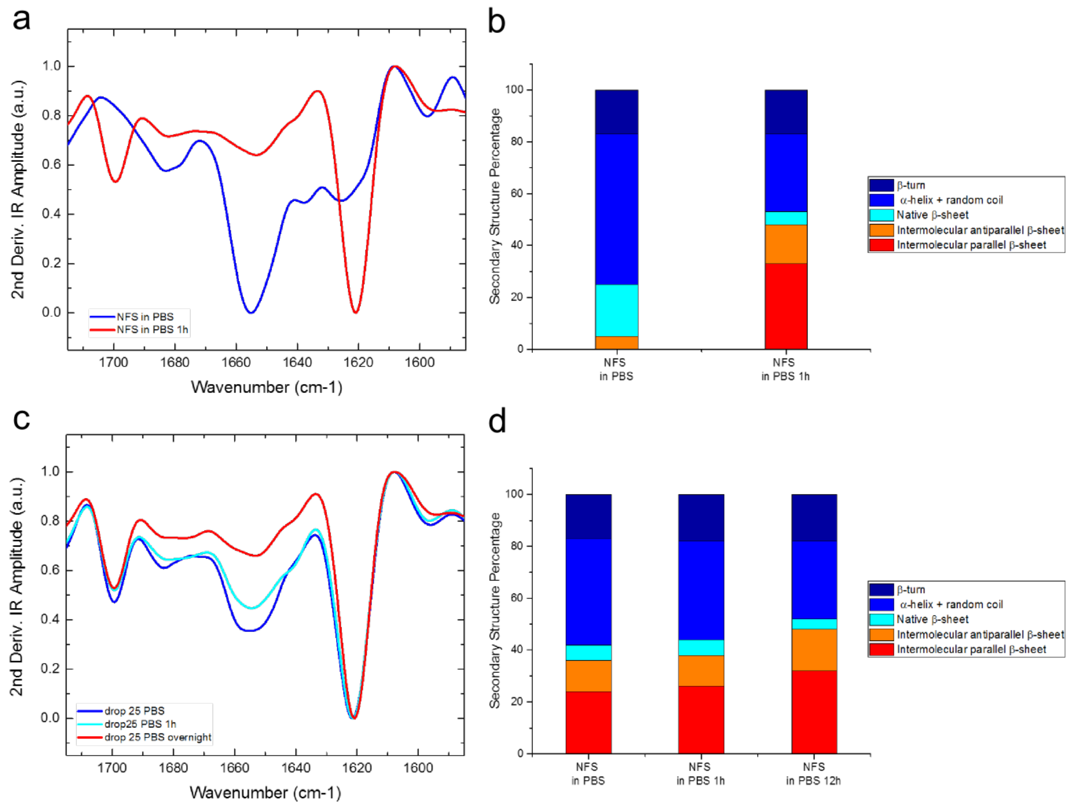

Supplementary Figure 6. (a) FTIR spectra indicating structural changes in soluble NSF exposed to phosphate buffer solution; for 0 s and 1 h. (b) Chart summarising calculations of the NSF fraction (in %) that retained its native confirmation after exposure to phosphate buffer solution for 0 s and 1 h. (c) FTIR spectra indicating structural changes in encapsulated NSF exposed to phosphate buffer solution; for 0 s, 1 h and 12 h. (d) Chart summarising changes in encapsulated NSF secondary structure after exposure to phosphate buffer. The calculations were made based on the difference in the amide I peak intensities in FTIR spectra from Supplementary Figure 6a and 6c.

The loading efficiency studies, performed systematically for the different morphologies, are summarised in Supplementary Figure 7a and 7c. For all the shapes, the loading efficiency exceeded 88%, demonstrating effective encapsulation and storage of small molecules. We found that we could tune the release kinetics by controlling the morphology of the micrococoons. The results show that the thick fibres exhibited the slowest release rate for remazol brilliant blue (RBBR), tetracycline and glucose and thin fibres possessed the fastest release rate, while spherical, cylindrical and short fibre structures displayed intermediate release kinetics. The results shown in Supplementary Figure 7b and 7d-f reveal marked differences in the rate of release of the different small molecules from the micrococoons. RBBR exhibited the slowest release rate for the all NSF micrococoon shapes and was not fully released even after one week, a result originating from the interaction of the dye molecule with the NSF. Behaviour similar to that of RBBR was observed for glucose. By contrast, the hydrophobic tetracycline antibiotic molecule reached its maximum release rate after 48 h. We performed further encapsulation (Supplementary Figure 7c) and release (Supplementary Figure 7d-f) experiments in phosphate buffer media (PBS). The results presented in Supplementary Figure 7d-f show almost no differences in the small molecule release rates in the buffer solution. For completeness we additionally verified, using MTS cell viability assays (Supplementary Figure 8), that the NSF micrococoon structures were not toxic to a human cell line.

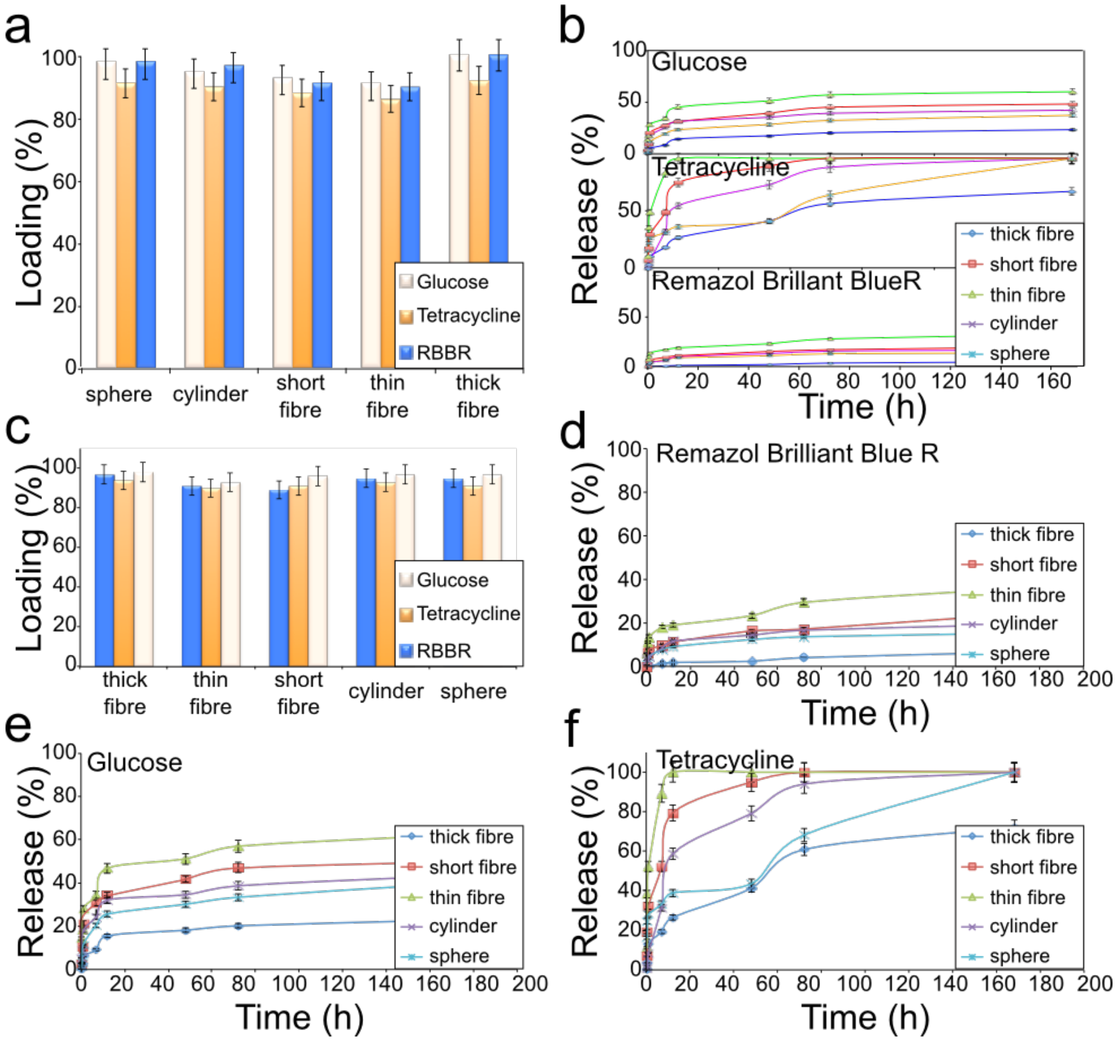

Supplementary Figure 7. (a) encapsulation efficiency studies for Remazol Brillian Blue R dye, tetracycline antibiotic and glucose molecules encapsulated in spherical, cylinder, short, thin and thick fibbers NSF micrococoon shapes in aqueous media. (b) release kinetics studies in aqueous media for Remazol Brillian Blue R dye, for glucose molecules and tetracycline antibiotic. (c) encapsulation efficiency studies for Remazol Brillian Blue R dye, tetracycline antibiotic and glucose molecules encapsulated in spherical, cylinder, short, thin and thick fibbers NSF micrococoons in PBS. (d) release kinetics studies in PBS for Remazol Brillian Blue R dye, (e) for glucose molecules and (f) tetracycline antibiotic. The indicated error bars are the standard deviation of the average of three different repeats, each one measure 10 times.

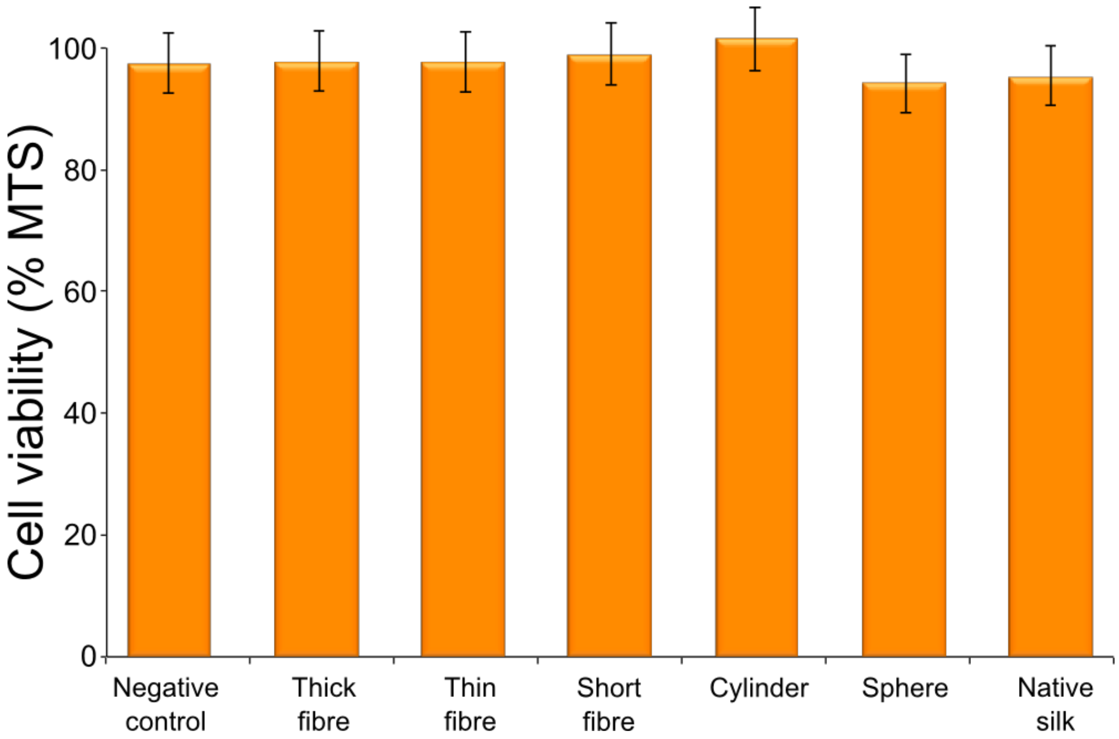

Supplementary Figure 8. Viability test with *SH-SY5Y* human cells. Cell viability was measured for the following solutions: NSF, NSF micrococoon shapes of: spheres, cylinders, short fibres, thin fibres, thick fibres and negative control. The indicated error bars are the standard deviation of the average of three different repeats.

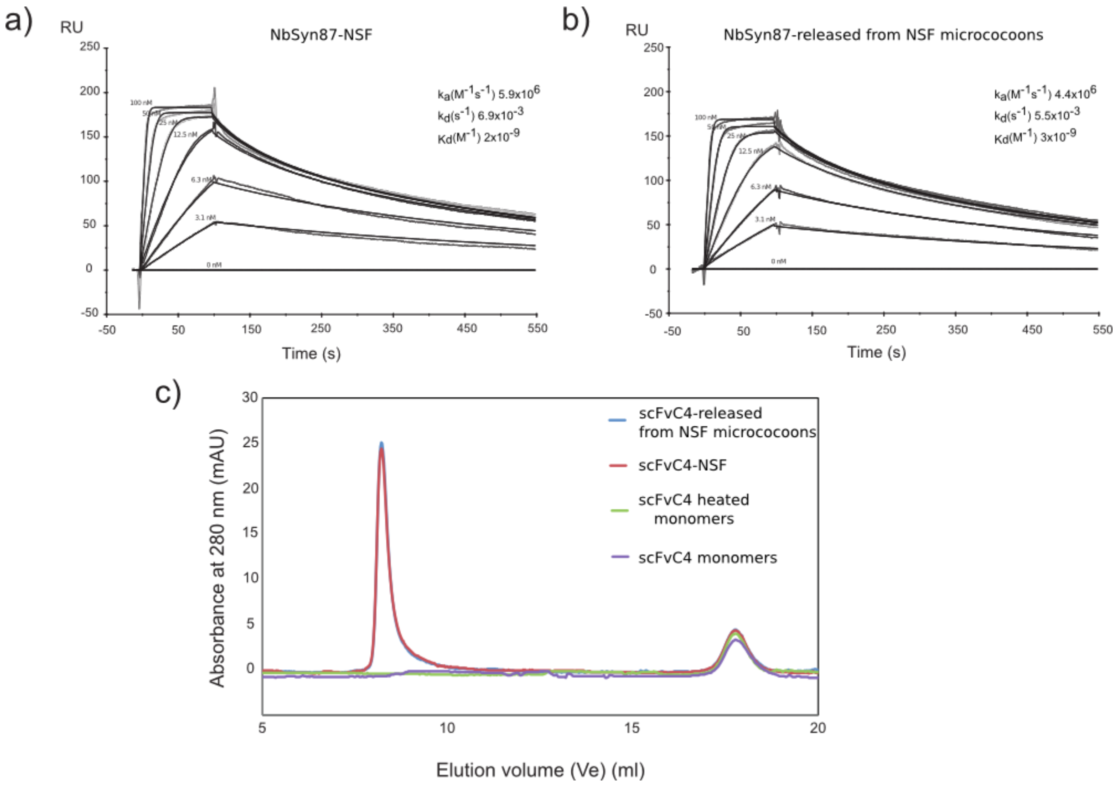

Supplementary Figure 9. (a) binding activity measurements for NbSyn87 mixed with NSF and (b) for NbSyn87 released from NSF micrococoons (See Methods). (c) size exclusion of C4scFv nanobodies, C4scFv nanobodies mixed with NSF dope and C4scFv nanobodies released from NSF micrococoons. The first absorbance maxima is attributed to the NSF content, while the second to nanobodies.

The partitioning of monomeric and fibrillar NSF in oil and aqueous phases was further examined under bulk conditions by means of two complementary optical methods, namely by BCA protein quantification of the soluble form (see Methods section in Supporting Information) and NSF intrinsic fluorescence-based quantification of the fibrillar protein form as shown in Supplementary Figure 10. The colorimetric BCA analysis revealed that 100% of the monomeric protein, at different concentrations (1, 5, 10 and 20 mg/ml), was found in the aqueous phase and 0% in oil phase, while the changes in partitioning of nanofibrillar protein in aqueous solution was observed when NSF fibrils concentration was increased above 10 mg/ml. It is worth to mention that the 0% of the fibrillar NSF was found in fluorinert oil. This increase in the NSF fibrillar content in aqueous phase resulted in the water/fibrilar NSF phase separation. The tendency for phase separation at high protein fibrils concentration is attributed to the hydrophobic nature of the NSF fibres.

NSF protein partitioning				
NSF concentration (mg/ml)	aqueous		oil	
	monomers (%)	fibrils (%)	monomers (%)	fibrils (%)
1	100	100	0	0
5	100	100	0	0
10	100	60	0	0
20	100	37	0	0

Supplementary Figure 10. NSF monomers and fibrils partitioning in oil and aqueous phases as a function of protein concentration.

Supplementary Methods

Native Silk Fibroin extraction from *B. mori* silkworm gland. Final instar *B. mori* were dissected and native silk fibroin extracted from the glands as described previously[50]. In short, final instar spinning *B. mori* silkworms had their silk glands extracted into DDW and the epithelium peeled between the postierior part of the middle division and the posterior part to extract gland contents with little to no sericin[51]. Gland contents were then placed into 2.5 ml microcentrifuge tubes and diluted to requirements with DDW. As *B. mori* silkworms are invertebrates, this study is in accordance with the Animals (Scientific Procedures) Act 1986 of the UK.

Atomic force microscopy. NSF micrococoons were cross-linked with 4% of paraformaldehyde solution (20 min incubation at 20°C), in order to keep the NSF structures intact, and then rinsed with a high concentration of aqueous ethanol solutions (20%, 50%, 70% and 100%). The micrococoons were deposited onto a mica slides and dried under ambient conditions, and then analysed and characterised by AFM microscopy using a H-02-0067 NanoWizard II (JPK Instrument).

AFM nanoindentation studies. PF-QNM measurements were performed by using a MultiMode VIII Scanning Probe Microscope (Bruker, USA) operated in intermittent mode either under ambient conditions or in a liquid environment at a scan rate of 1 Hz. The microscope was covered with an acoustic hood to minimise vibrational noise. The AFM cantilevers were calibrated on defined samples (Bruker, USA) covering the following ranges of Young's moduli: from 100 MPa to 2 GPa (for low-density polyethylene) and from 1 to 20 GPa (for polystyrene). The analysis of the Derjaguin–Mueller–Toporov (DMT) modulus was performed using Nanoscope Analysis software.

Silk release studies. The release of NSF from the inner content of the spherical form of micrococoons was achieved by either a fast-freeze method (liquid nitrogen), gentle centrifugation (700 rpm, 3 min) or incubation of the NSF micrococoons in aqueous media. The degree of aggregation for the released NSF was measured by FTIR spectroscopy, following the change in the beta-sheet vibration band. The experiments were repeated 5 times each.

Small molecule encapsulation efficiency and release kinetics studies. For studies of encapsulation and release kinetics, aqueous solutions of 0.01% of Remazol Brilliant BlueR (RBBR) dye, tetracycline antibiotic and glucose solution (Sigma-Aldrich, UK) were used. For encapsulation studies the RBBR dye (0.01% w/v), tetracycline and glucose (Sigma-Aldrich, UK) components was dissolved in aqueous protein media (NSF dissolved in water). Glucose was quantified using a glucose assay kit (Sigma-Aldrich, UK). The loading capacity and release kinetics of small molecules from NSF micrococoons was studied by following the change in absorption maxima at 592 nm for RBBR, 360 nm for tetracycline and 540 nm for glucose by using a glucose detection kit according to the manufacture's protocol (See manufacture protocol for glucose detection kit in Sigma-Aldrich). For encapsulation and release efficiency studies more than 10 repeated experiments were performed (10 replicated samples were measured 3 times each). In order to study the release kinetics of NSF.

micrococoons, the loaded samples were washed with DDW at pH 7 within following intervals of time: 10 min, 30 min, 1 h, 3 h, 7 h, 12 h, 24 h, 48 h, 3 d, 7 d, 10 d. Each washing solution was then analysed by UV-spectroscopy and the exact concentration of the released molecules was detected.

Shear stress. The shear stress for the NSF micrococoon formation inside the microfluidic channels was calculated by multiplying the viscosity of the aqueous NSF solution[14] and the shear rate (τ = μ'γ). The shear rate for the fluids in the microdroplet device was approximated by the maximum flow velocity divided by the device height ('γ ~υ/h).

FTIR analysis. Structural analysis of the NSF micrococoons was performed by using an FTIR- Equinox 55 spectrometer (Bruker). The samples (washed with DDW) were used without further pretreatment, and were loaded into the FTIR holder and analysed by subtracting a water reference. The atmospheric compensation spectrum were subtracted from the original FTIR spectra and a secondary derivative was applied for further analysis. The small differences in FTIR spectra of NSF are due to the extraction of NSF from different worms; these difference do not impact on the interpretation of results. Each FTIR measurement was repeated 3 times for every sample replicate (in average 12 replicates per sample). The sensitivity of the instrument was detected to be 5%. To resolve the transformation of native structure of NSF into aggregated fibrils, we followed the vibrational changes in amide I, which is strictly correlated with protein secondary structure. The FTIR band assignment with NSF secondary structure is shown in Supplementary Figure 3a.

Structural changes in NSF and NSF microdroplets under de-stabilising conditions. The structural changes in NSF in bulk and in microdroplets were studied by following the changes in corresponding FTIR spectra. The samples were exposed to the following destabilising conditions: 1) shaking (300 rpm for 5 s, 10 s, 15 s, 20 s, 25 s, 1 h, 12 h and 48 h) , 2) temperature (25°C, 30°C, 40°C, 50°C and 65°C) and 3) PBS buffer (for 5 s, 1 h and 12 h).

Fluorescence spectroscopy analysis. The fluorescence spectra of native and aggregated NSF in bulk and in gels was monitored by fluorescence spectroscopy using a Cary Eclipse fluorescence spectrophotometer. The samples were pre-scanned to calculate the excitation and emission maximum. The emission maxima were determined by exciting samples at wavelengths varying from 300 nm to 415 nm with intervals of 5 nm each scan. The excitation maximum was detected by measuring spectrum at fixed emission wavelengths varying from 400 nm to 515 nm with an interval of 5 nm for each scan.

Cell viability assays. *SH-SY5Y* human neuroblastoma cells were incubated in 96-well plates with 100 μl of following NSF micrococoon shapes for 24 h at 37°C: 1) NSF; 2) NSF spheres; 3) cylinders; 4) short fibres; 5) thin fibres; 6) thick fibres. After 24 h in culture in Opti-MEM Reduced Serum Medium (Gibco, UK) cell viability was measured using CellTiter 96 Aqueous One Solution Cell Proliferation Assay (Promega, UK), and a plate reader (Fluostar Optima, BMG Labtech, UK).

Expression, purification and labeling of antibody fragments. The antibody fragment, NbSyn87, was previously isolated through phage display selection following the immunization of a lama with the A53T variant of human α−Syn, and the expression and purification of NbSyn87 was performed according the protocol described in the same paper[52]. To obtain Alexa-Fluor 647 labeled NbSyn87, we mixed a solution of 70~nmol of NbSyn87 with 1.5 equivalent of Alexa Fluor 647-succinimidyl ester (Life Technologies, Paisley, UK) in 1 ml of 100 mM sodium carbonate buffer, pH9.0. The reaction mixture was then incubated in the dark and at room temperature (RT) for 5 h. After the reaction, the free dye was separated from the labeled protein using a PD10 desalting column, containing 8.3 ml of Sephadex 25 resin (GE-Healthcare, Little Chalfont, UK). The labelling yield and stoichiometry were determined by absorbance spectrophotometry. The nanobody, NbSyn86, was obtained from the same phage-display library that yielded NbSyn87 and though identical selection strategies as described previously[52]. NbSyn86 was also expressed and purified in an identical way to that described for NbSyn87. The cloning expression and purification of the C4scFv protein will be

described elsewhere (De Genst et al; in preparation). Labeling of C4scFv with Alexa-fluor647 was obtained in an identical way to that described for NbSyn87.

Analytical gel filtration experiments. To evaluate the conformational integrity of C4scFv upon release after encapsulation by micrococoons, we performed size exclusion experiments using a Superdex 200 (10/30) high resolution gel filtration column (GE Heathcare). Samples of C4scFv of 100 μl volume were injected onto the column using an Akta basic instrument (GE Healthcare). Elution profiles were recorded for one column volume by measuring the absorbance at 280 nm.

Encapsulation of antibody fragments in NSF micrococoons. The loading and release efficiency studies of the antibody fragments from NSF micrococoons were probed on fluorescently labeled with AlexaFluor647 dye C4scFv antibody domain. 70 nmol of labeled fragment were mixed with an aqueous NSF solution prior to microfluidic processing. The AlexaFluor647-C4scFv loaded NSF micrococoons were formed by following the standard protocol which described in Methods section (See Droplet microfluidics). After formation, the were washed with PBS at intervals of time from 10 min to 30 d, and the solutes after each washing were analysed by UV/vis and fluorescence spectroscopy.

Activity measurements of NbSyn86 and NbSyn87 using surface plasmon resonance. The NbSyn87 binding activity was measured using surface plasmon resonance using a Biacore 2000 (GE Healthcare) instrument. One flow-cell of a CM5 sensor chip (GE Healthcare) containing 250 RU of immobilised α−synuclein and a blank control flow-cell, were prepared using EDC/NHS amine coupling chemistry according to the manufactures recommendations. A concentration range of NbSyn87, corresponding to initial concentrations ranging from 0 nM to 100 nM was then prepared in HBS-EP running buffer pH7.4 (GE Healthcare, UK) for each sample containing NbSyn87 and subsequently injected for 2 min onto the sensor chip at a flow rate of 30 μl/min to follow the association of the binding reactions. The dissociation of the nanobody in the running buffer at a flow-rate of 30 μl/min was followed for 10 min immediately after injection. The kinetic curves were fitted to a binding model that accounts for mass-transport effects, implemented in the BIA evaluation software (GE Healthcare, UK).
The binding measurements involving NbSyn86 were performed using a Biacore 3000 instrument and a CM5 sensor chip that was coated with 150 RU of α−synuclein using amine coupling chemistry. The preceding flow-cell served again as a blank reference surface. From all samples used in the encapsulation experiments that contained NbSyn86, a series of concentrations were prepared ranging from 0 nM to 200 nM. Kinetic traces were recorded as described above for the NbSyn87 samples. The kinetics of the interaction were, however, too fast to be measured accurately. Therefore the equilibrium binding levels were measured and plotted against the initial concentration of NbSyn86. Fitting of these data to a Langmuir binding model yielded an estimate of the Kd of the interaction for each sample.

Measurements of NSF protein partitioning in water and oil solutions. The equal volumes of fluorinert oil and aqueous NSF solutions, containing different protein concentrations of 1, 5, 10, 20 mg/ml, were mixed up in the reaction epindorph, followed by incubation for 24 h at 4°C, in order to achieve complete phase separation. The NSF monomers partitioning in aqueous and oil phases was studied using BCA protein assay kit (ThermoFisherScientific) set to measure (A562 nm) total protein concentration compared to a protein standard. The NSF fibres partitioning in oil and in water solutions was detected by measuring NSF intrinsic fluorescent signal with emission maxima at 450 nm.
